# A cross-sectional study of current and lifetime sexual hallucinations and delusions in Lebanese patients with schizophrenia: frequency, characterization, and association with childhood traumatic experiences and disease severity

**DOI:** 10.1186/s12888-022-04012-z

**Published:** 2022-05-27

**Authors:** Sarah Gerges, Chadia Haddad, Tracy Daoud, Christina Tarabay, Mikhael Kossaify, Georges Haddad, Souheil Hallit

**Affiliations:** 1 School of Medicine and Medical Sciences, Holy Spirit University of Kaslik, P.O. Box 446, Jounieh, Lebanon; 2 Research Department, Psychiatric Hospital of the Cross, Jal Eddib, Lebanon; 3 INSPECT-LB (Institut National de Santé Publique, d’Épidémiologie Clinique et de Toxicologie-Liban), Beirut, Lebanon; 4 School of Health Sciences, Modern University of Business and Science, Beirut, Lebanon; 5 Psychology Department, College of Humanities, Effat University, Jeddah, 21478 Saudi Arabia

**Keywords:** Schizophrenia, Psychotic disorders, Sexual trauma, Emotional abuse, Lebanon

## Abstract

**Background:**

Till that date, a sparse body of research has been dedicated to perusing psychotic symptoms of sexual type, particularly in psychiatric populations. Our study’s objective was to delineate psychotic symptoms with a sexual content, namely sexual delusions and hallucinations, among inpatients diagnosed with schizophrenia in Lebanon, and scrutinize their relationships with the severity of schizophrenia symptoms and childhood abusive events.

**Methods:**

We conducted structured interviews with 167 chronic schizophrenia patients, who completed the Questionnaire for Psychotic Symptoms with a Sexual Content, the Child Abuse Self-Report Scale, and the Positive and Negative Syndrome Scale.

**Results:**

36.5% and 50.3% of the participants screened positive for current and lifetime episodes of sexual delusions and/or hallucinations, respectively. Alcohol drinking (aOR (adjusted odds ratio)_current_ = 2.17; aOR_Lifetime_ = 2.86) and increased psychological (aOR_current_ = 1.09; aOR_Lifetime_ = 1.09) and sexual (aOR_current_ = 1.23; aOR_Lifetime_ = 1.70) abuse were significantly associated with higher chances of experiencing current and lifetime sexual hallucinations and/or delusions. Additionally, an increased severity of schizophrenia symptoms (aOR = 1.02) was significantly associated with higher chances of current sexual hallucinations and/or delusions, whereas having a university level of education compared to primary (aOR = 0.15) was significantly associated with lower odds of current sexual hallucinations and/or delusions.

**Conclusion:**

In sum, our findings suggest that sexual psychotic symptoms are prevalent in chronic schizophrenia patients, providing support for their associations with antecedents of childhood traumatic experiences, illness severity, and substance use disorders. They endorse the vitalness of preventive measures against abuse, in order to circumvent such phenomenological outcomes. Our study offers the first data on sexual hallucinations and delusions in a non-Western psychiatric population, thus allowing clinicians and researchers to draw featural comparisons across different cultural settings.

## Background

Schizophrenia is a serious mental disorder regularly encountered during clinical practice. In point of fact, reported estimates have suggested a lifetime prevalence of schizophrenia varying between 0.28% and 0.7%, notwithstanding a vast dissimilitude between countries and ethnicities [1, 2]. This disease is characterized by chronic psychotic symptomatology (i.e., delusions and hallucinations), disorganized speech/behavior, negative symptoms (e.g., emotion suppression), as well as substantial social and occupational impairments [1], thus contributing to innumerable life years burdened with disability worldwide [2].

Although researchers have steadily improved their understanding of delusions and hallucinations (e.g., verbal, auditory, etc.) in schizophrenia-spectrum psychotic disorders [3, 4], using both phenomenological and analytical approaches, till that date, a sparse body of research has been dedicated to perusing psychotic symptoms of sexual type, particularly in psychiatric populations. Indeed, auditory and visual hallucinations were the most frequently documented in schizophrenia psychosis [5]. However, among all modalities of hallucinations (i.e., auditory, visual, olfactory, gustatory, and tactile), sexual types have been the least analyzed so far [6]. In fact, despite several reports describing the presence of sexual hallucinations and delusions within psychotic disorders [7, 8], particularly among schizophrenia patients [9–14], only two precedent studies have considered elaborating on their potential psychopathological correlates [6, 15].

Precisely, an Australian study examining the presence of psychotic symptoms of a sexual nature among subjects at high risk for psychosis, namely genetically predisposed populations, showed that 15.2% of the patients manifested psychotic symptoms with an explicit sexual connotation, while 35.9% had a past history of sexual molestation or rape [15]. Interestingly, among those who reported sexual trauma, 33% experienced sexual psychosis and no less than 45.5% had at least one delusional symptom of being watched or followed [15]. Consistently, within another study conducted among patients diagnosed with a schizophrenia spectrum disorder in the Netherlands, the totality of participants reporting sexual hallucinations detained a past history of childhood traumatic events, with a vast predominance of the sexual form (76.9%) [6].

Remarkably, the latter research underlined significant levels of perceived distress among over two thirds of hallucinating patients. In reality, those patients made plain their aversion to hallucinatory episodes of a sexual nature, describing a wide set of unpleasant emotions, including shame, despair, rage, fear, and helplessness [6]. Indeed, Blom and Mangoenkarso have emphasized the extremely deleterious psychosocial outcomes of these conditions in patients with schizophrenia spectrum disorders, ranging from substantial discomforts to completed suicide. Clinical experience evidences that schizophrenia patients might perceive sexual visual illustrations, sounds, tastes and/or odors, feel sexually metamorphosed, or incur somatic sexual processes, such as inappropriate arousal and/or orgasmic sensations. As a result, these phenomena unquestionably disrupt their psychological and social functioning [6]. Consequently, although not the integrity of sexual psychotic symptoms has come in line with despicable impairments [16], paramount attention must be paid to these understudied conditions, urging health care providers and investigators to establish proficient interventions, in furtherance of preserving and protecting vulnerable populations.

Nevertheless, the body of research exploring these entities remain surprisingly tenuous, even absent, among a multitude of nations, and particularly in oriental cultural backgrounds. Since the presence and contents of delusional and hallucinatory disorders are immensely modulated by individuals’ cultural, socioeconomic and religious backgrounds [1], it has gotten fascinating to rigorously investigate this subject in Lebanon, a collectivist and conservative society. Therefore, our study’s objective was to delineate psychotic symptoms with a sexual content, namely sexual delusions and hallucinations, among inpatients diagnosed with schizophrenia in Lebanon—a non-Western developing country—and scrutinize their relationships with the severity of schizophrenia symptoms and childhood abusive events. In light of the aforementioned facts, we hypothesized that precedent traumatic childhood experiences would intensify sexual psychosis among schizophrenia patients [6, 15]. Additionally, in referral to current evidence that indicates the tight link between unmanaged psychosis and symptom severity [17, 18], we expected that sexual delusions and hallucinations would be more potentiated if the symptoms of schizophrenia were more severe.

## Methods

### Study design

**Fig. 1:**
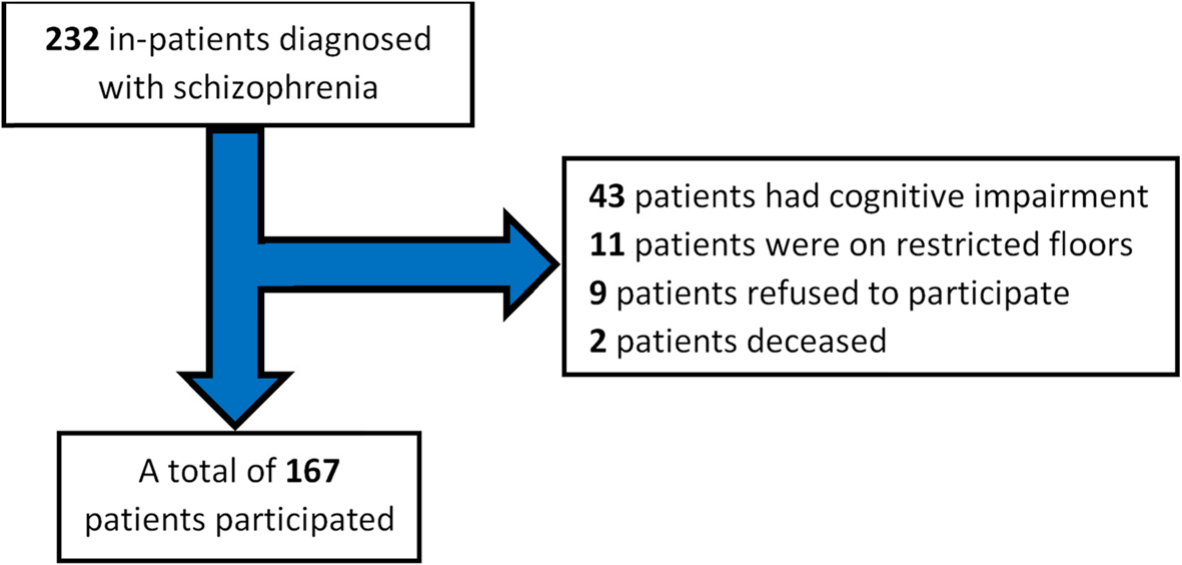
Flow chart of the participants

During the month of February 2022, healthcare professionals, overseen by clinical psychiatrists, conducted structured face-to-face interviews with 167 patients diagnosed with schizophrenia. The diagnosis should have been made by two clinical psychiatrists, in referral to the Diagnostic and Statistical Manual – 5^th^ Edition (DSM-5) criteria [1]. All the interrogated patients were chronically hospitalized (i.e., for a 1-year period or more) at the Psychiatric Hospital of the Cross, Lebanon. In addition, all the participants were aged 18 years or over. We excluded patients diagnosed as cognitively impaired according to the Mini-Mental State Exam and thus unable to respond properly, as well as those who refused to participate, and those who were on restricted floors due to the COVID-19 pandemic (Fig. 1).

### Sample description

Table 1 presents the sociodemographic and other characteristics of the patients.

**Table 1:** Sociodemographic and other characteristics of the participants (*N* = 167)

Categorical Variables	N (%)
**Gender**	
Male	98 (58.7%)
Female	69 (41.3%)
**Marital Status**	
Single/divorced/widowed	157 (94.0%)
Married	10 (6.0%)
**Education Level**	
Primary	57 (34.1%)
Complementary	50 (29.9%)
Secondary	45 (26.9%)
University	15 (9.0%)
**Religion**	
Christian	107 (64.1%)
Muslim	60 (35.9%)
**Illegal Drug Use**	
Yes	12 (7.2%)
No	155 (92.8%)
**Smoking Status**	
Yes	101 (60.5%)
No	66 (39.5%)
**Alcohol Use**	
Yes	46 (27.5%)
No	121 (72.5%)
**Continuous Variables**	**Mean ± SD**
**Age**	56.10 ± 12.29
**Duration of Treatment (in years)**	16.94 ± 12.20
**Duration of Hospitalization (in years)**	12.62 ± 10.98
**Cumulative Cigarette Smoking**	260.62 ± 433.24

### Minimal sample size calculation

Relying on the work of Blom and Mangoenkarso, which revealed that patients who had experienced sexual abuse were 8.7 times more likely to have psychotic symptoms of a sexual nature [6], the Epi info software version 7.2 [19] indicated that including a minimum of 36 participants was compulsory to reach significance, with a statistical power of 0.8 and an acceptable alpha error of 0.05. Ultimately, 167 of the hospitalized patients diagnosed with schizophrenia were eligible for inclusion.

### Translation procedure

Except for the Arabic version of Positive and Negative Syndrome Scale (PANSS), which has been previously validated in Lebanon [20], the used scales in this study were translated into Arabic, Lebanon’s native language, then back-translated into English by two distinct healthcare professionals. The back-translated English versions were compared to the measures’ original English versions, in furtherance of detecting and dismissing any incongruity. This forward–backward translation process was perpetuated until all the discordances were solved, in conformance with international guidelines for an adequate cross-cultural adaptation of health assessment instruments [21].

### Questionnaire and variables

We performed individualized interviews with the eligible patients, in the Arabic language, under the supervision of clinical psychiatrists. The mean duration of each questioning was 15 min. We first collected data about their sociodemographic characteristics (i.e., age, gender, marital state, level of education, and religion) as well as their total years of treatment and hospitalization, based on their medical files. Patients were asked about their substance use habits (i.e., alcohol use (Yes/No), illegal drug use (Yes/No), and smoking status (Yes/No)). We computed the cumulative cigarette smoking exposure, multiplying the number of smoked cigarettes per day by the number of smoking years. The second part of the interview consisted of standardized measures, as follows:

#### The Questionnaire for psychotic symptoms with a sexual content (Sexual delusions and hallucinations)

This tool is a semi-structured questionnaire, which was purposely compiled for a previous study targeting sexual psychosis in schizophrenia spectrum disorders [6]. It consists of 27 items that assess the presence and nature/characteristics of sexual delusions and sexual hallucinations. Responses are categorized into “absent”, “currently present” (i.e., during the past two weeks), and “previously present” (N.B. the latter response was added in the present study to check for a past history of delusions/hallucinations and allow screening for lifetime occurrences). Some questions also require free-text answers, such that patients might explicitly describe the specific phenomenology of their sexual psychotic symptoms [6]**.** In this study, the Cronbach’s alpha was 0.797 for sexual delusions, 0.794 for sexual hallucinations, and 0.869 for the total questionnaire.

#### ***The Child Abuse ***Self***-Report Scale (CASRS)***

This instrument includes 38 statements that evaluate the presence and frequency of numerous facets of childhood adversities, namely psychological abuse (through 14 items), parental neglect (through 11 items), physical maltreatment (through 8 items), and sexual trauma (through 5 items). Response options range from “never happened” (scored as 0) to “always” (scored as 3). Greater scores denote an amplified severity of child abuse [22]. The Cronbach’s alpha values were as follows: 0.945 for psychological abuse, 0.943 for physical abuse, for 0.910 sexual abuse, 0.938 for neglect, and 0.921 for the whole scale.

#### ***The Positive and ***Negative*** Syndrome Scale (PANSS)***

This measure represents a succession of 30 symptoms that fall under schizophrenia disease, including seven positive symptoms (i.e., delusions, conceptual disorganization, hallucinatory behavior, excitement, grandiosity, suspiciousness, and hostility), seven negative symptoms (i.e., blunted affect, emotional withdrawal, poor rapport, passive-apathetic social withdrawal, difficulty in abstract thinking, lack of spontaneity and flow of conversation, and stereotyped thinking), and sixteen symptoms belonging to the general psychopathology of schizophrenia illness (i.e., somatic concern, anxiety, guilt feelings, tension, mannerisms and posturing, depression, motor retardation, uncooperativeness, unusual thought content, disorientation, poor attention, lack of judgment and insight, disturbance of volition, poor impulse control, preoccupation, and active social avoidance). The severity of symptoms is coded on a 7-point Likert scale, varying from the total “absence” of a particular symptom (scored as 1) to an “extremely severe” manifestation (scored as 7). Higher scores reflect an escalation of schizophrenia symptoms (i.e., increased disease gravity) [23]. This scale has also proved its validity and reliability among Lebanese patients diagnosed with schizophrenia [20]. In the present study, Cronbach’s alpha = 0.746 for the positive scale, 0.719 for the negative scale, 0.833 for the general psychopathology scale, and 0.869 for the entire scale.

### Statistical analysis

We used the SPSS software version 22 for the statistical analysis. As all the questions were mandatory, we retrieved no missing data. For reliability analysis, we calculated the Cronbach’s alpha of each scale and its subscales. For comparing two means, the Student t test was used. For comparing two categorical variables, the Chi-square test was applied (the Fischer test was used when there is *n* < 5 in a cell). Then, we conducted two logistic regressions to investigate the predictive variables associated with the presence of current and lifetime sexual hallucinations and/or delusions, including all the independent variables that had a p-value inferior to 0.25 in the bivariate analyses [24]. Given the cross-sectional design of our study, the current PANSS score is inapt to predict lifetime sexual hallucinations and/or delusions; consequently, this variable was only included in the analyses of current episodes of sexual psychosis. Statistical significance was judged achieved for *p*-values < 0.05 in the bivariate analyses and final multivariate model.

## Results

### Frequency and characterization of sexual hallucinations and delusions among our sample

Details about sexual hallucinations and delusions among our sample are displayed in Table 2.

**Table 2:** Frequency and Description of Sexual Hallucinations and Delusions among our sample

	**Current Episodes**	**Past Episodes**	**Lifetime Screening**^**a**^
**Sexual Delusions and/or Hallucinations**	**61 (36.5%)**	**39 (23.4%)**	**84 (50.3%)**
**Sexual Delusions**	53 (31.7%)	29 (17.4%)	72 (43.1%)
Delusions of being spied	28 (16.8%)	6 (3.6%)	34 (20.4%)
Jealousy or Infidelity from a Loved One	13 (7.8%)	9 (5.4%)	22 (13.2%)
Sexual Metamorphosis	14 (8.4%)	3 (1.8%)	17 (10.2%)
Change of Size/Shape of Genital Organs	22 (13.2%)	9 (5.4%)	31 (18.6%)
Delusions of being Persecuted for Sexual Reasons	11 (6.6%)	3 (1.8%)	14 (8.4%)
Delusions regarding Sexual Behaviors	16 (9.6%)	4 (2.4%)	20 (12.0%)
Delusions regarding Body Parts/Movements with a Sexual Connotation	13 (7.8%)	4 (2.4%)	17 (10.2%)
Delusions of Being Pregnant	8 (4.8%)	5 (3.0%)	13 (7.8%)
**Sexual Hallucinations**	35 (21.0%)	22 (13.2%)	51 (30.5%)
Tactile Hallucinations	14 (8.4%)	3 (1.8%)	17 (10.2%)
Visual Hallucinations	11 (6.6%)	3 (1.8%)	14 (8.4%)
Auditory Hallucinations	8 (4.8%)	4 (2.4%)	10 (6.0%)
Olfactory Hallucinations	7 (4.2%)	1 (0.6%)	8 (4.8%)
Gustatory Hallucinations	6 (3.6%)	0 (0%)	6 (3.6%)
Genital Hallucinations	15 (9.0%)	9 (5.4%)	24 (14.4%)
Somatic Hallucinations	27 (16.2%)	13 (7.8%)	39 (23.4%)

^a^ Lifetime screening includes any current or past episode of sexual hallucinations/delusions

Among patients who reported psychotic symptoms of sexual type, some provided further qualitative descriptions. To exemplify, one male patient had delusions of having become a eunuch (i.e., having been castrated), due to the implantation of a spying machine inside his body by “unknown enemies”, in the form of a voluminous kidney stone. He held the conviction that this electronic spy device was recording his words and movements while keeping him sterile; therefore, he was secretly begging his doctor to give him a diuretic, in order to get rid of that kidney stone. A female participant believed she was adored by a Federal Bureau of Investigation (FBI) female special agent named “Freud”, who was willing to deliver her from the hospital. The agent used to visit her virtually (via her “spirit”) several times a week at night. Additionally, the patient had sexual tactile hallucinations and orgasmic sensations when “Freud” used to apply a blue laser over her body, thus procuring a sexual-like tingling sensation in all her body parts. Another female patient had daily nocturnal auditory hallucinations, as she was hearing voices inciting her to masturbate all night. Several patients reported visual hallucinations of a particular abuser throughout their lives.

### Bivariate analysis: correlates of sexual hallucinations and/or delusions

Table 3 displays the bivariate analysis taking the presence of sexual hallucinations and/or delusions: 1-current episodes and 2-lifetime screening as the dependent variables. The results showed that a significantly higher proportion of illegal drug users and alcohol users reported current and lifetime sexual hallucinations and/or delusions. In addition, significantly higher mean total PANSS, positive PANSS, and general psychopathology PANSS scores were found among those reporting current sexual hallucinations and/or delusions as compared to those who did not manifest these symptoms. Moreover, significantly higher mean psychological, physical, and sexual abuse scores were found among those reporting current and lifetime sexual hallucinations and/or delusions compared to those who did not.

**Table 3:** Bivariate analysis taking current and lifetime sexual hallucinations and/or delusions as dependent variables

	**Current**		***p*-value**	**Lifetime**		***p*-value**
	**Presence of Sexual Hallucinations and/or Delusions**	**Absence of Sexual Hallucinations and/or Delusions**		**Presence of Sexual Hallucinations and/or Delusions**	**Absence of Sexual Hallucinations and/or Delusions**	
**Categorical Variables**	**N (%)**	**N (%)**		**N (%)**	**N (%)**	
**Gender**						
Male	39 (39.8%)	59 (60.2%)	0.296	52 (53.1%)	46 (46.9%)	0.395
Female	22 (31.9%)	47 (68.1%)		32 (46.4%)	37 (53.6%)	
**Marital Status**						
Single/divorced/widowed	58 (36.9%)	99 (63.1%)	0.658	78 (49.7%)	79 (50.3%)	0.527
Married	3 (30.0%)	7 (70.0%)		6 (60.0%)	4 (40.0%)	
**Education Level**						
Primary	24 (42.1%)	33 (57.9%)	0.195	30 (52.6%)	27 (47.4%)	0.653
Complementary	20 (40.0%)	30 (60.0%)		23 (46.0%)	27 (54.0%)	
Secondary	15 (33.3%)	30 (66.7%)		25 (55.6%)	20 (44.4%)	
University	2 (13.3%)	13 (86.7%)		6 (40.0%)	9 (60.0%)	
**Religion**						
Christian	36 (33.6%)	71 (66.4%)	0.302	54 (50.5%)	53 (49.5%)	0.954
Muslim	25 (41.7%)	35 (58.3%)		30 (50.0%)	30 (50.0%)	
**Illegal Drug Use**						
Yes	10 (83.3%)	2 (16.7%)	** < 0.001**	10 (83.3%)	2 (16.7%)	**0.018**
No	51 (32.9%)	104 (67.1%)		74 (47.7%)	81 (52.3%)	
**Smoking Status**						
Yes	38 (37.6%)	63 (62.4%)	0.716	53 (52.5%)	48 (47.5%)	0.487
No	23 (34.8%)	43 (65.2%)		31 (47.0%)	35 (53.0%)	
**Alcohol Use**						
Yes	24 (52.2%)	22 (47.8%)	**0.010**	33 (71.7%)	13 (28.3%)	**0.001**
No	37 (30.6%)	84 (69.4%)		51 (42.1%)	70 (57.9%)	
**Continuous Variables**	**Mean ± SD**	**Mean ± SD**		**Mean ± SD**	**Mean ± SD**	
**Age**	55.75 ± 12.03	56.30 ± 12.49	0.783	55.21 ± 12.09	57.00 ± 12.50	0.350
**Duration of Treatment (in years)**	15.88 ± 12.13	17.55 ± 12.25	0.397	16.13 ± 11.72	17.75 ± 12.68	0.392
**Duration of Hospitalization (in years)**	12.44 ± 11.12	12.72 ± 10.94	0.874	12.32 ± 10.80	12.91 ± 11.21	0.729
**Cumulative Cigarette Smoking**	195.09 ± 291.14	298.33 ± 494.35	0.091	229.10 ± 350.99	292.51 ± 503.22	0.246
**Total PANSS scale**	69.00 ± 24.47	52.55 ± 19.13	** < 0.001**			
Positive PANSS	18.29 ± 8.56	12.36 ± 7.00	** < 0.001**			
Negative PANSS	15.11 ± 7.28	13.32 ± 6.38	0.100			
General Psychopathology PANSS	35.59 ± 15.24	26.86 ± 10.97	** < 0.001**			
**Psychological Abuse**	8.86 ± 10.58	2.50 ± 5.08	** < 0.001**	7.52 ± 9.56	2.09 ± 5.17	** < 0.001**
**Physical Abuse**	3.31 ± 5.48	1.30 ± 3.01	**0.010**	3.00 ± 4.98	1.06 ± 2.90	**0.003**
**Sexual Abuse**	2.22 ± 3.90	0.54 ± 1.38	**0.002**	2.05 ± 3.53	0.24 ± 0.80	** < 0.001**
**Neglect**	12.90 ± 8.62	10.93 ± 9.24	0.177	12.45 ± 8.53	10.84 ± 9.51	0.252

Numbers in bold indicate significant *p*-values

### Multivariate analysis

The first logistic regression showed that alcohol drinking (aOR (adjusted odds ratio) = 2.17), increased severity of schizophrenia symptoms (i.e., higher PANSS scores; aOR = 1.02), and increased psychological (aOR = 1.09) and sexual (aOR = 1.23) abuse (i.e., higher CASRS scores) were significantly associated with higher chances of experiencing current sexual hallucinations and/or delusions, whereas having a university level of education compared to primary (aOR = 0.15) was significantly associated with lower odds of current sexual hallucinations and/or delusions.

The second logistic regression showed that alcohol drinking (aOR = 2.86) and increased psychological (aOR = 1.09) and sexual (aOR = 1.70) abuse were significantly associated with higher chances of screening positive for lifetime sexual hallucinations and/or delusions (Table 4).

**Table 4:** Logistic regression analysis taking current and lifetime sexual hallucinations and/or delusions as dependent variables

	**Current**		**Lifetime**	
	**aOR (95% CI)**	***p*-value**	**aOR (95% CI)**	***p*-value**
Education Level (University vs Primary^a^)	0.15 (0.02–0.86)	**0.033**		
Alcohol Use (yes vs no^a^)	2.17 (1.12–4.17)	**0.020**	2.86 (1.46–5.60)	**0.002**
Cumulative Cigarette Smoking	0.99 (0.99–1.00)	0.083	0.99 (0.99–1.00)	0.090
Total PANSS score	1.02 (1.00–1.04)	**0.005**		
Psychological Abuse	1.09 (1.02–1.17)	**0.008**	1.09 (1.02–1.17)	**0.009**
Physical Abuse	0.98 (0.87–1.11)	0.819	1.04 (0.91–1.18)	0.595
Sexual Abuse	1.23 (1.02–1.49)	**0.029**	1.70 (1.22–2.37)	**0.002**
Neglect	0.98 (0.93–1.03)	0.509		

Numbers in bold indicate significant *p*-values. Variables not presented in the table did not show significance

*aOR* Adjusted odds ratio, *CI* Confidence interval

^a ^Reference group

## Discussion

Within this study, we worked towards building on the scarce body of knowledge addressing sensitive and understudied conditions (i.e., sexual psychotic symptoms) among schizophrenia patients. Interestingly, our findings highlighted high occurrences of current (36.5%) and lifetime (50.3%) sexual hallucinations and/or delusions in this clinical population. Specifically, 31.7% and 21% of our sample had current episodes of sexual delusions and sexual hallucinations, respectively. Consistently, far back in 1962, Lucas et al. depicted delusions with sexual contents in 126 out of 405 patients with an established diagnosis of schizophrenia, yielding a prevalence rate of 31.1% in their chronic psychotic sample [25]. Additionally, in 1983, Lyketsos et al. recorded sexual hallucinations in 19.5% of chronic schizophrenia patients [26]. Conversely, recent studies have come across much lower incidences. To specify, Bloom and Mangoenkarso retrieved a 1-year prevalence of 1.67% for sexual hallucinations among patients with schizophrenia spectrum disorders [6], while Thompson et al. witnessed the presence of psychotic symptoms with sexual contents among 15.2% of the screened patients with high risk for psychosis [15]. However, unlike the current research targeting chronic psychiatric individuals with an established psychotic disorder, the latter studies’ results were drawn from observations of broader psychiatric conditions (i.e., schizophrenia spectrum, brief limited intermittent psychotic symptoms, etc.), hence justifying those disparities. Further, given the high interference of culture with the prevalence and nature of experienced psychotic symptoms [1], it is plausible that these phenomena are more prevailing among the Lebanese population than in European countries. Further research in other Eastern and Arab countries is warranted to test this conjecture and advocate our findings.

A conspicuous outcome of the present study was the association of sexual psychotic beliefs and symptoms with antecedents of child emotional and sexual abuse. Certainly, within the literature, childhood adversities have been strongly inferred as potent risk factors for the development of psychotic manifestations (i.e., hallucinations and delusions) [27, 28], specifically in schizophrenia disorder [29]. Those symptoms have been presumed to serve as defensive coping strategies, instantiating aversive childhood recalls by dysfunctional sensory phenomena [30]. On another hand, the socio-developmental-cognitive theory suggests that childhood traumatic events enhance internal distress that results in deregulated dopamine synthesis and release, which, together with cognitive processes biased by social adversity, lead to the induction and perpetuation of psychosis [31]. To exemplify, Sheffield et al. demonstrated that childhood abuse, specifically the sexual-type, heightens the chances of auditory hallucinations in psychotic disorder patients [32]. Further, during the last century, Beck and van der Kolk have documented greater rates of sexual delusions in chronic psychotic patients who had confronted childhood incest, highlighting as well the correlation of abuse with the severity and intractability of psychotic disorders [33]. Moreover, recent empirical evidence has lent support for the association of prior sexual abuse with delusions/hallucinations of sexual type within a broad spectrum of psychotic disorders [6, 15]. Yet, to the best of our knowledge, this study is the first to sustain this relationship in a clinical sample of chronic schizophrenia patients. Nonetheless, the strength of this discussed association was observed to be higher in previous research (computed odds ratios of 7.31 to 8.7 [6, 15] versus 1.23 in the current study). Those irregularities may be attributed to the abovementioned differences in the studied psychotic populations, as well as the relatively small samples used to conduct the previous analyses, in comparison with the present study.

This study underlined the relationship between majored scores on the positive and negative syndrome scale, denoting an increased severity of schizophrenia illness, and current sexual hallucinations and/or delusions. Indeed, researchers have realized that psychosis was related to more severe forms of schizophrenia disease [17]. On another hand, prior research has also identified exacerbated schizophrenia symptoms and elevated psychosis severity in patients reporting a history of trauma/abuse [33–35]. These facts might suggest a common path linking child abuse, the extent of severity in schizophrenia pathology, and psychotic symptoms, especially those with sexual contents. Future longitudinal studies exploring this paradigm would be useful to test the temporality of events and provide insights into the outcomes of abuse throughout the long course of chronic schizophrenia.

Furthermore, within our sample, alcohol drinking was significantly associated with an escalated susceptibility for episodes of sexual hallucinations and delusions, corroborating the work of previous researchers. In fact, available data posits that patients presenting a psychotic disorder, including schizophrenia, with concomitant substance abuse and/or dependence—a condition referred to as dual diagnosis—are more vulnerable to psychotic symptoms when compared to patients without such comorbidities [36]. Our study thus postulates that this dual diagnosis’ effect holds true for sexual psychotic symptoms as well. Hence, given the highly prevailing comorbidity between schizophrenia and substance use disorders [1, 37], health care providers must strive to detect and treat those disorders in schizophrenia patients, as a means to attenuate psychotic symptoms and assuage their deleterious impacts on mental health.

In the last place, regarding social characteristics, patients with a higher level of education were less likely to have sexual hallucinations and delusions. This result is in agreement with a previous study conducted among male schizophrenia patients in Saudi Arabia, which spotlighted a greater severity of psychotic symptoms in participants with lower educational levels (i.e., basic/primary education) [38]. Our finding speculates that patients having reached fewer academic levels would probably suffer a shortfall of functional coping competencies [39] and thus enhance sexual hallucinations and delusions as maladaptive compensatory mechanisms for their mental distress.

### Clinical implications

This research expands our knowledge into the cognitive contents molding hallucinations and delusions experienced in a frank psychotic illness, that is schizophrenia, thus shedding light on the dysfunctional cognitive schemas held by this population. From this point derive paramount clinical implications. Indeed, characterizing sexual hallucinations/delusions would help complement the promising psychotherapeutic approaches for psychotic disorders, which are essentially built on cognitive therapy [40–42]. Our findings also give prominence to the utility of screening patients with sexual psychosis for a past history of abuse and concomitant substance use disorders. Those factors must be effectively targeted in clinical practice; hence, this study underscores the need to implement national intervention programs against abuse and rigorous therapeutic regimens aiming to decrease the severity and progression of schizophrenia disease, in order to prevent the development of aversive psychosis of sexual type among this specific population.

### Strengths and limitations

To our best knowledge, our study is the first non-Western scrutinization of sexual hallucinations/delusions among patients with schizophrenia, and it is the sole of its kind to demonstrate their relationships with alcohol drinking, educational level, and disease severity, while reinforcing the results of previous Western investigations that have accentuated the potential involvement of childhood trauma in the psychopathology of sexual psychotic symptoms. Nonetheless, our study also presents some limitations. As the gathered data only captures a snapshot in time, causality and temporality of the established associations cannot be inferred. As a result, this study’s findings must be interpreted with caution. These associations might also have been modulated by further residual determinants not included in the current study. In addition, the findings relied on interviews with patients, who might have been uncomfortable reporting such phenomena and misreported their symptoms, leaving our results susceptible to a plausible information bias. In this context, even while precedent investigations have numerously shown that individuals with psychotic disorders can be considered reliable in recalling their childhood adversities [43, 44], questions covering childhood trauma predispose our findings to a recall bias. Lastly, we recruited the participants from a single psychiatric hospital, limiting the aptitude to generalize our findings to all patients diagnosed with schizophrenia in Lebanon.

## Conclusion

In sum, our findings suggest that sexual psychotic symptoms are prevalent in chronic schizophrenia patients, providing support for their associations with antecedents of childhood traumatic experiences, illness severity, and substance use disorders. They endorse the vitalness of preventive measures against abuse, in order to circumvent such phenomenological outcomes. Our study offers the first data on sexual hallucinations and delusions in a non-Western psychiatric population, thus allowing clinicians and researchers to draw featural comparisons across different cultural settings. Further research with higher levels of analysis are warranted to better understand the biopsychosocial mechanisms governing these associations.

## Data Availability

All data generated or analyzed during this study are not publicly available to maintain the privacy of the individuals’ identities. The dataset supporting the conclusions is available upon request to the corresponding author.
